# Comparison of Diagnostic Yield Between Fine Needle Aspiration Cytology and Core Needle Biopsy in the Diagnosis of Thyroid Nodule

**DOI:** 10.3390/diagnostics15202566

**Published:** 2025-10-11

**Authors:** Yeongrok Lee, Myung Jin Ban, Do Hyeon Kim, Jin-Young Kim, Hyung Kwon Byeon, Jae Hong Park

**Affiliations:** 1 Department of Otolaryngology Head and Neck Surgery, Soonchunhyang University College of Medicine, Cheonan 31151, Republic of Korea; yso_sirius@naver.com (Y.L.); mjbanent@gmail.com (M.J.B.); rlaehgus199570@gmail.com (D.H.K.); 2 Division of Respiratory Allergy and Critical Care Medicine, Department of Internal Medicine, Soonchunhyang University College of Medicine, Cheonan 31151, Republic of Korea; 109947@schmc.ac.kr; 3 Department of Otolaryngology Head and Neck Surgery, Soonchunhyang University College of Medicine, Seoul 04401, Republic of Korea

**Keywords:** thyroid nodule, ultrasonography, fine needle aspiration cytology, core needle biopsy

## Abstract

**Background/Objectives**: This study aimed to evaluate the effectiveness of core needle biopsy (CNB) by comparing its diagnostic yield to fine needle aspiration cytology (FNAC) across primary and secondary examinations. **Methods**: This retrospective review analyzed medical records of patients who visited Soonchunhyang University Cheonan Hospital between January 2021 and August 2023 for thyroid nodule evaluation. Demographic data and the malignancy risk of thyroid nodules were collected based on the 2021 Korean Thyroid Imaging Reporting and Data System. FNAC and CNB results, classified using the Bethesda system for reporting thyroid cytopathology and diagnostic categories for thyroid CNB, were categorized as either “conclusive” or “inconclusive.” The rates of conclusive results in the primary examination and nodules transitioning from inconclusive to conclusive results during the secondary examination were analyzed. Finally, the diagnostic yields of FNAC and CNB were assessed using histopathological findings from surgically excised nodules. **Results**: The rate of nodules classified as “conclusive” was significantly higher in the CNB group than that in the FNAC group. Among nodules subjected to secondary examination, only the group with FNAC followed by CNB demonstrated a significant improvement in the rate of transition from inconclusive to conclusive results. Although FNAC and CNB showed comparable sensitivity and accuracy, the specificity of CNB was greater than that of FNAC. **Conclusions**: This study confirms the clinical utility of CNB by demonstrating its higher rate of conclusive results than FNAC. Future prospective studies, including cost–benefit analyses, are warranted to further define the indications for CNB.

## 1. Introduction

Fine-needle aspiration cytology (FNAC) is widely recommended for diagnosing thyroid nodules owing to its simplicity and cost-effectiveness [1,2,3]. However, the incidence of inconclusive cytology results, such as non-diagnostic outcomes or atypia of undetermined significance/follicular lesion of undetermined significance (AUS/FLUS), is regarded as its main shortcoming [4]. In such cases, repeated FNAC is often required to establish a definitive diagnosis [5]. Unfortunately, the rates of non-diagnostic or AUS/FLUS results in consecutive FNAC examinations remain substantial, ranging from 17% to 47% overall [6,7,8,9] and reaching up to 67% in nodules with initially inconclusive findings [10,11,12,13].

To address the limitations of FNAC, core needle biopsy (CNB) has been introduced as an alternative diagnostic approach for thyroid nodules. Numerous studies have demonstrated that CNB is more effective than repeated FNAC in achieving definitive diagnoses for nodules initially classified as non-diagnostic by FNAC [14,15,16,17,18]. Additionally, recent studies have validated the efficacy of CNB as a primary diagnostic procedure for thyroid nodules. Despite these findings, most studies have [19,20,21] focused either on CNB’s utility as a primary diagnostic method or on its effectiveness as a secondary diagnostic test for nodules deemed non-diagnostic by FNAC.

In this study, we aimed to evaluate the efficacy of CNB by comparing the diagnostic accuracy of FNAC and CNB when used as primary and secondary diagnostic tools, respectively.

## 2. Materials and Methods

### 2.1. Patients

This retrospective study, based on the analysis of medical records, was approved by the Institutional Review Board of Soonchunhyang University Cheonan Hospital (SCHCA 2024-07-023, The date of approval was 9 August 2024). The study included 289 patients who visited the Department of Otorhinolaryngology-Head and Neck Surgery at Soonchunhyang University Cheonan Hospital and underwent FNAC or CNB for thyroid nodule evaluation between January 2021 and August 2023. All patients were informed of the potential complications, costs, and respective advantages and disadvantages of FNAC and CNB before selecting their preferred diagnostic method and providing informed consent. Before undergoing FNAC or CNB, all patients received high-resolution ultrasonography (Alpinion E-CUBE 15, Alpinion Medical Systems^®^, Seoul, Republic of Korea) to evaluate the number, size, margins, and internal echogenicity of each nodule. The risk of malignancy in the thyroid nodules was assessed according to the revised 2021 guidelines of the Korean Society of Thyroid Radiology (K-TIRADS). FNAC or CNB was performed when pathological confirmation was required [3,22]. Two experienced otorhinolaryngologists, each with over seven years of experience in ultrasonography, conducted all procedures. FNAC was performed for large cystic nodules, small nodules located lateral or posterior to major vessels, and in cases where needle or biopsy device access was restricted to the transverse plane of the ultrasound probe. In all other cases, the type of diagnostic test was determined based on patient preference. During the study period, FNAC was performed on 248 thyroid nodules, and CNB was performed on 111 thyroid nodules.

### 2.2. Evaluation of Ultrasonographic Findings

**Figure 1:**
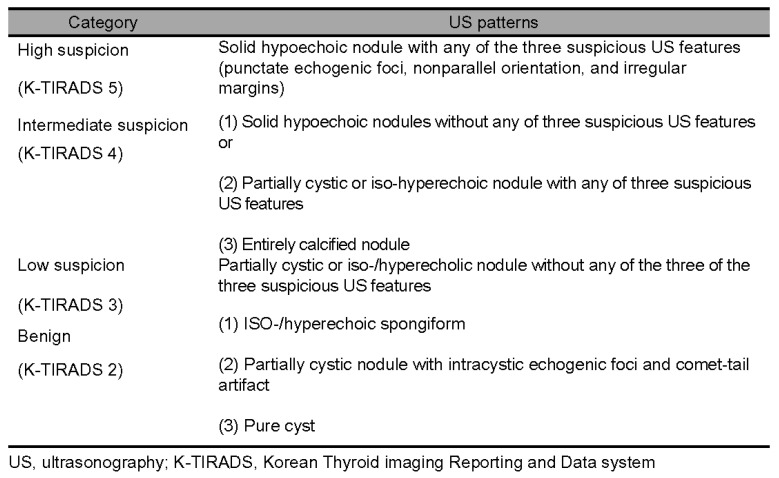
Illustration of the 2021 Korean Thyroid Imaging Reporting and Data System (2021 K-TIRADS) [22].

All thyroid nodules were retrospectively analyzed according to the revised 2021 K-TIRADS, regardless of the examination period. The analysis assessed the composition, echogenicity, orientation, margin, and echogenic foci of the nodules. Ultrasonographic features suggestive of malignancy, including non-parallel orientation, irregular margins, and punctate echogenic foci within solid components smaller than 1 mm, were categorized into four K-TIRADS categories ranging from 2 to 5 (Figure 1) [22]. Nodules classified as K-TIRADS 2 were excluded from biopsy recommendations per the guidelines. Consequently, 20 thyroid nodules initially evaluated by FNAC and one nodule evaluated by CNB due to a recent size increase were excluded from the analysis.

### 2.3. Fine Needle Aspiration Cytology

**Figure 2:**
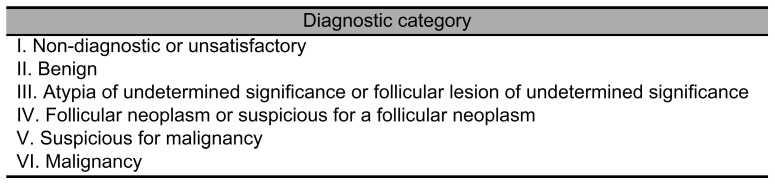
The Bethesda System for Reporting Thyroid Cytopathology (Bethesda system) [5].

FNAC was performed with the patient in a supine position, with the neck extended using a pillow placed under the shoulders. Under high-resolution ultrasound guidance, a 23-gauge needle was inserted into the lesion, and cellular material was aspirated through repeated back-and-forth motions. The aspirated samples were smeared onto glass slides and fixed in 95% alcohol. The procedure was repeated 1 to 3 times to ensure adequate sample collection. For pure cystic nodules, the needle was inserted, and the contents were aspirated to the maximum extent possible in a single attempt. These samples were fixed using liquid-based cytology. The fixed specimens were stained with Papanicolaou stain and examined under a light microscope. Diagnoses were made according to the Bethesda system for reporting thyroid cytopathology, which classifies samples into six diagnostic categories (Figure 2) [5].

### 2.4. Core Needle Biopsy

**Figure 3:**
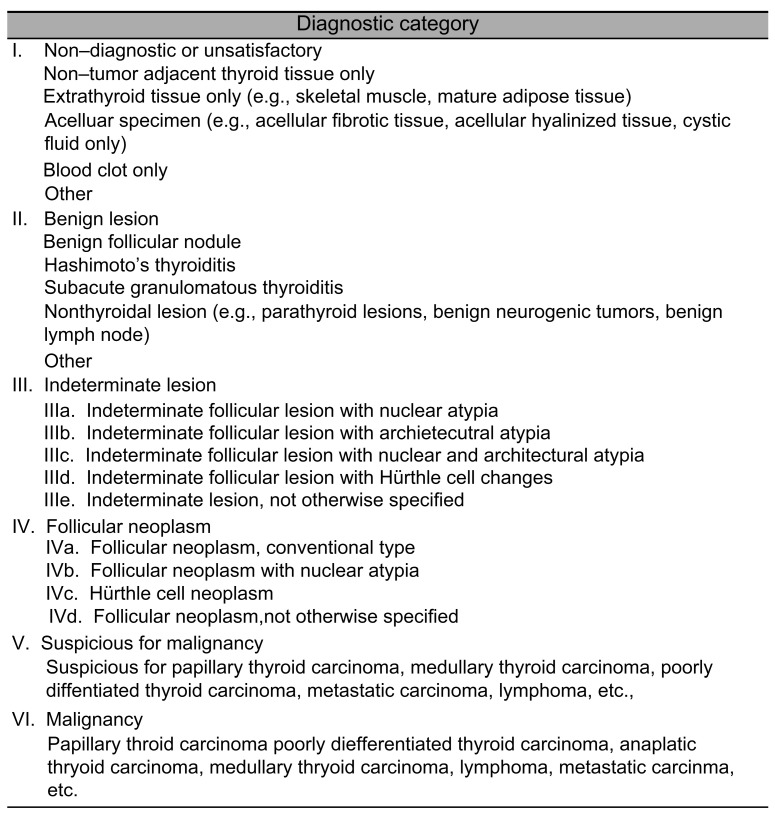
Diagnostic categories of thyroid core needle biopsy suggested by the 2019 Practice Guidelines of the Korean Thyroid Association [23].

Similar to the FNAC procedure, an ultrasound examination was conducted, followed by the administration of local anesthesia using 2% lidocaine with 1:100,000 epinephrine at the skin and anticipated needle path. A TSK Ace-cut device (Create Medic^®^, Yokohama, Japan) with an 11 mm advance length and 18-gauge thickness was used for the biopsy. Under ultrasound guidance, the biopsy device was inserted parallel to the transducer’s transverse axis. The tip of the device was positioned at the boundary of the lesion or 2–3 mm away. The stylet and cutting cannulas were fired sequentially to collect the sample, with only one pass per lesion to minimize the risk of bleeding. The obtained samples were fixed in 10% formalin and classified into six diagnostic categories based on the 2019 Practice Guidelines for Thyroid Core Needle Biopsy by the Korean Thyroid Association (Figure 3) [23].

### 2.5. Classification of Primary Examination Results

The diagnostic categories outlined in the Bethesda system and the 2019 practice guidelines for thyroid core needle biopsy of the Korean Thyroid Association were unified, as both systems classify results into non-diagnostic (Category I), benign (Category II), AUS/FLUS (Category III), follicular neoplasm/suspicious for follicular neoplasm (FN/SFN) (Category IV), suspicious for malignancy (Category V), and malignancy (Category VI). The outcomes of primary FNAC and CNB were categorized as either “conclusive” (Categories II, IV, V, and VI), which could determine further management without additional testing, or “inconclusive” (Categories I and III), which required further diagnostic procedures. The diagnostic yield of FNAC and CNB was compared by analyzing the frequency of “conclusive” and “inconclusive” results for each test type. Among patients with conclusive diagnoses from the initial tests, those confirmed with malignancy or follicular neoplasm, as well as those with compressive symptoms due to the size of the masses, were recommended for surgical intervention.

### 2.6. Classification of Secondary Examination Results

Among the nodules that underwent primary FNAC, those with inconclusive results or those confirmed as benign but classified as K-TIRADS 5 on ultrasound were subjected to secondary FNAC or CNB. Based on the secondary test performed, the nodules were divided into two groups: FNAC/FNAC (primary FNAC followed by secondary FNAC) and FNAC/CNB (primary FNAC followed by secondary CNB). The diagnostic outcomes for each group were categorized as ‘conclusive’ or ‘inconclusive’ according to the Bethesda System and the 2019 Practice Guidelines for Thyroid Core Needle Biopsy of the Korean Thyroid Association. The conversion rates from inconclusive primary results to conclusive secondary results were compared between groups. Nodules that underwent multiple tests following primary FNAC were classified according to the final test performed.

**Figure 4:**
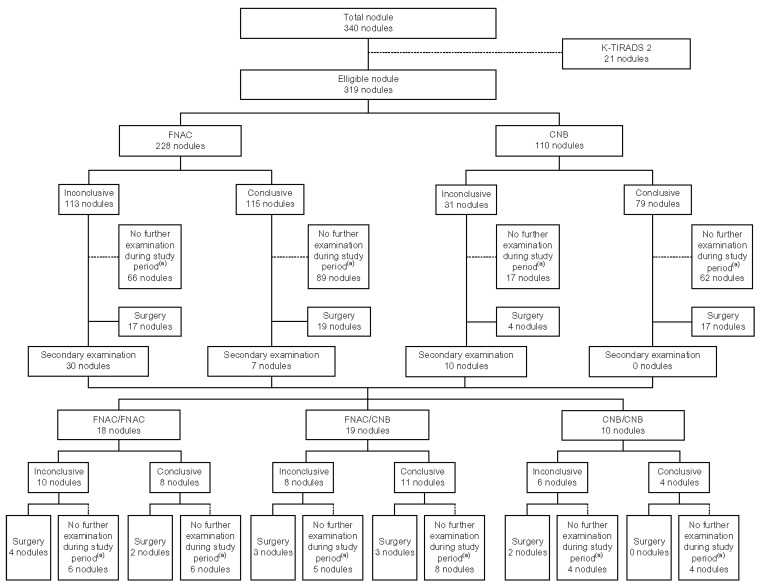
Study flowchart. ^(a)^ Reasons for no further examination included loss to follow-up, routine ultrasonographic evaluation only, and referral to another hospital owing to patient preferences. A conclusive result is defined as Category II (benign), Category IV (follicular neoplasm/suspicious for follicular neoplasm), Category V (suspicious for malignancy), and Category VI (malignancy) according to the Bethesda system and 2019 Practice Guidelines for Thyroid Core Needle Biopsy of the Korean Thyroid Association. An inconclusive result is defined as Category I (non-diagnostic) and Category III (atypia of undetermined significance/follicular lesion of undetermined significance) according to the Bethesda system and 2019 Practice Guidelines for Thyroid Core Needle Biopsy of the Korean Thyroid Association. FNAC: Fine-needle aspiration cytology; CNB: Core needle biopsy. Of the nodules included, 19 underwent both FNAC and CNB simultaneously; therefore, the total number of unique nodules is 319.

Similarly, nodules that initially underwent CNB and yielded inconclusive results or those confirmed as benign but classified as K-TIRADS 5 on ultrasound were subjected to secondary testing and categorized into the CNB/CNB group. CNB results were classified as ‘conclusive’ or ‘inconclusive’ according to the 2019 Practice Guidelines for Thyroid Core Needle Biopsy by the Korean Thyroid Association. If the results remained in Category III after two CNB tests, surgical excision was recommended. Other surgical indications remained consistent with those following the primary test (Figure 4).

### 2.7. Comparison of Diagnostic Yield Between FNAC and CNB Based on Postoperative Definitive Histopathological Evaluation

**Figure 5:**
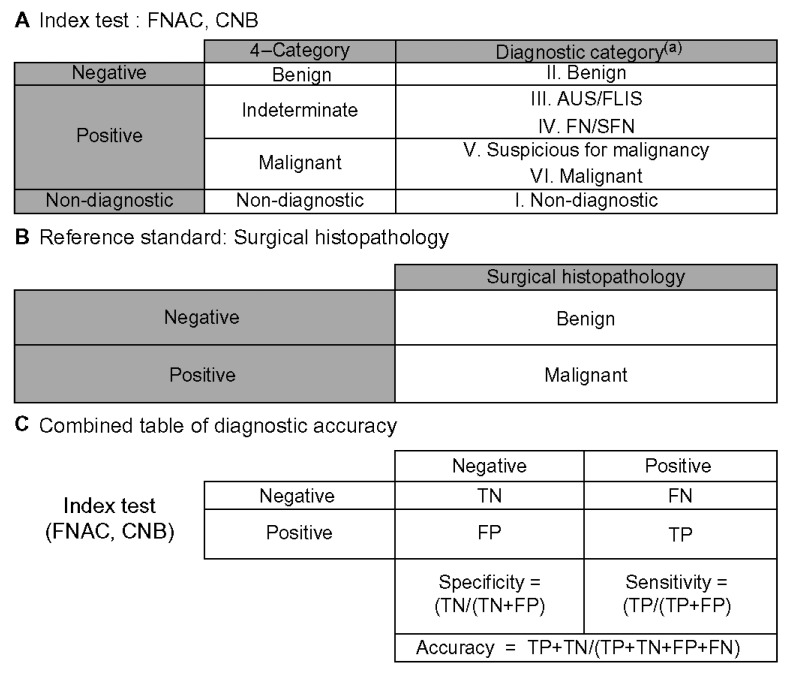
Schematic for calculating diagnostic accuracy [24]. (**A**) Benign fine-needle aspiration cytology (FNAC) or core needle biopsy (CNB) results were considered negative. All FNAC and CNB results that were neither benign nor inconclusive were classified as positive FNAC or CNB results. (**B**) Reference standard consisted solely of surgical histopathology. (**C**) A 2 × 2 table of diagnostic accuracy was constructed using the above definitions. ^(a)^ Diagnoses were categorized according to the six categories of the Bethesda system and the 2019 Practice Guidelines for Thyroid Core Needle Biopsy of the Korean Thyroid Association. FNAC: Fine-needle aspiration cytology; CNB: Core needle biopsy; AUS/FLUS: atypia of undetermined significance/follicular lesion of undetermined significance; FN/SFN: follicular neoplasm/suspicious for follicular neoplasm; FN: false-negative; FP: false-positive; TN: true-negative.

The diagnostic yield of the final preoperative FNAC and CNB samples was analyzed using the histopathological diagnosis obtained after surgery. Based on the Bethesda System and the 2019 Practice Guidelines for Thyroid Core Needle Biopsy by the Korean Thyroid Association, Category I was classified as non-diagnostic, Category II as benign, Categories III and IV as indeterminate, and Categories V and VI as malignant. The final preoperative results were reclassified into these four categories, and the sensitivity, specificity, and accuracy of FNAC and CNB were calculated (Figure 5) [24].

### 2.8. Statistical Analysis

The evaluation of primary diagnostic tests was performed using the chi-square test or Fisher’s exact test for categorical data and Student’s *t*-test for continuous data. The McNemar test was employed to compare the conversion rates of inconclusive results to conclusive results in secondary tests across different groups. Statistical analyses were conducted using IBM SPSS 23.0 statistical software (IBM Corp., Armonk, NY, USA), with a *p*-value of less than 0.05 considered statistically significant.

## 3. Results

### 3.1. Patient Demographic and Ultrasonographic Findings

A total of 228 nodules from 196 patients underwent primary FNAC, and 110 nodules from 93 patients underwent primary CNB. Among the patients in the primary FNAC group, 64 (32.7%) were men and 132 (67.3%) were women. In the primary CNB group, 28 (30.1%) patients were men and 65 (69.9%) were women, with no statistically significant difference in sex distribution between the two groups (*p* > 0.05). The mean ages of the patients in the primary FNAC and CNB groups were 54.3 ± 15.4 and 56.9 ± 15.2 years, respectively, showing no statistically significant difference (*p* > 0.05).

The mean size of nodules on ultrasound prior to biopsy was 1.80 ± 0.61 cm in the primary FNAC group and 1.72 ± 1.10 cm in the primary CNB group, with no statistically significant difference between the groups (*p* > 0.05). When analyzing the K-TIRADS results of the thyroid nodules categorized as low-, intermediate-, and high-suspicion, 99 (43.4%), 84 (36.9%), and 45 (19.7%) nodules in the primary FNAC group were identified as low-, intermediate-, and high-suspicion, respectively. In the primary CNB group, 50 nodules (45.5%) were classified as low suspicion, 28 (25.5%) as intermediate suspicion, and 32 (29.0%) as high suspicion. There was no statistically significant difference in the overall distribution between the groups (*p* = 0.054) (Table 1).

### 3.2. Primary Examination Results

A total of 228 nodules underwent FNAC, and 110 nodules underwent CNB during the study period. Nineteen nodules were evaluated by both methods simultaneously, making the total number of eligible nodules 319.

The number of nodules diagnosed as Category I (non-diagnostic), Category II (benign), Category III (AUS/FLUS), Category IV (FN/SFN), Category V (suspicious for malignancy), and Category VI (malignancy) after primary testing were as follows: in the primary FNAC group, there were 68 (29.8%), 92 (40.4%), 45 (19.7%), 1 (0.4%), 19 (8.3%), and 3 (1.3%) nodules, respectively. In the primary CNB group, there were 3 (2.7%), 58 (52.7%), 28 (25.5%), 2 (1.8%), 9 (8.2%), and 10 (9.1%) nodules, respectively. When classified as ‘conclusive’ and ‘inconclusive,’ the primary FNAC group had 115 (50.4%) conclusive and 113 (49.6%) inconclusive results, whereas the primary CNB group had 79 (71.8%) conclusive and 31 (28.2%) inconclusive results. The difference in the proportion of conclusive and inconclusive results between the two groups was statistically significant (*p* < 0.05) (Table 2).

**Figure 6:**
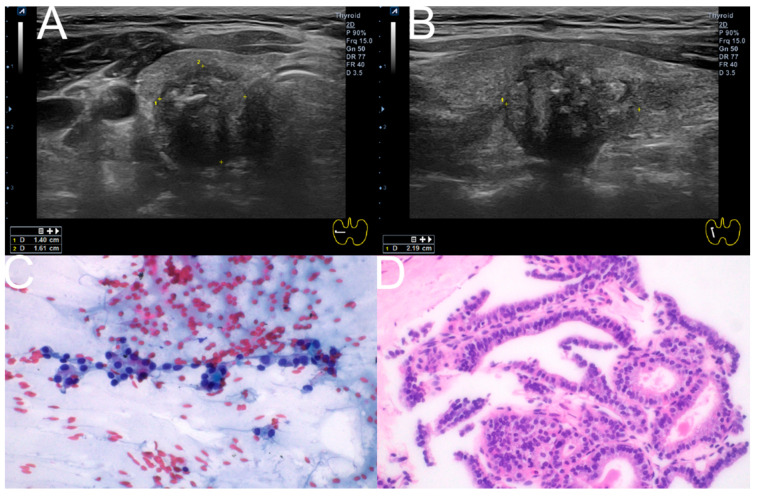
Composite illustration of the diagnostic pathway for a representative case. (**A**,**B**) Ultrasonographic images (Transverse, longitudinal) of the right thyroid showing a 1.40 × 1.61 × 2.19 cm hypoechoic nodule with irregular margins and macrocalcification, classified as K-TIRADS 5. (**C**) FNAC smear (Papanicolaou stain, ×400) demonstrating features corresponding to Bethesda Category III (AUS/FLUS). (**D**) CNB specimen (hematoxylin and eosin stain, ×400) from the same nodule, classified as Category V according to the 2019 Practice Guidelines for Thyroid Core Needle Biopsy of the Korean Thyroid Association. FNAC: Fine-needle aspiration cytology; CNB: Core needle biopsy; AUS/FLUS: atypia of undetermined significance/follicular lesion of undetermined significance.

Among the primary tests, 19 nodules underwent both CNB and FNAC simultaneously, with eight showing discrepant results between the two tests. Of these, FNAC results were inconclusive for seven nodules. Surgery was performed on five of these seven nodules, revealing two benign nodules and three cases of papillary thyroid carcinoma (Table 3) (Figure 6).

### 3.3. Secondary Examination Results

Among the 144 nodules initially diagnosed as inconclusive, 40 underwent secondary testing. Additionally, out of 14 nodules initially diagnosed as conclusive but meeting the criteria for secondary testing, 7 underwent secondary testing, resulting in a total of 47 nodules. Of the seven nodules with conclusive results that underwent repeat testing, three were benign nodules with K-TIRADS 5 features on ultrasound, and the remaining four were repeated for other reasons. Of these, 37 nodules underwent FNAC and 10 underwent CNB as secondary tests. In the FNAC/FNAC group, 14 (77.8%) of the 18 were initially diagnosed as inconclusive, and 6 (33.3%) converted to conclusive upon secondary testing. Conversely, two of the four nodules (11.1%) initially diagnosed as conclusive were reclassified as inconclusive in the secondary test. Overall, there was no statistically significant difference in the conversion rates between the initial and secondary test results (*p* > 0.05) (Table 4). Among the eight nodules (44.4%) that remained inconclusive in both tests, three underwent surgery, revealing two cases of papillary thyroid carcinoma and one benign nodule.

In the FNAC/CNB group, 16 of 19 nodules (84.2%) were initially diagnosed as inconclusive, with eight nodules (42.1%) converting to conclusive upon secondary testing. None of the three nodules initially diagnosed as conclusive were reclassified as inconclusive in the secondary test. The conversion rates between the initial and secondary test results showed a statistically significant difference (*p* < 0.05) (Table 5). Among the eight nodules (42.1%) that remained inconclusive in both tests, three underwent surgery, revealing one case of papillary thyroid carcinoma, one case of Hashimoto’s thyroiditis, and one benign nodule.

In the CNB/CNB group, all 10 nodules were initially diagnosed as inconclusive, with four nodules (40%) converting to conclusive results upon secondary testing. Owing to the small sample size, the analysis of conversion rates between the initial and secondary tests was limited. Among the six nodules (60%) that remained inconclusive in both tests, two underwent surgery, revealing two benign nodules.

### 3.4. Comparison of Diagnostic Yields Between FNAC and CNB Based on Post-Surgical Definitive Histopathological Results

Thyroidectomy was performed on 62 nodules based on the results of the primary and secondary tests. Histopathological analysis revealed 36 cases of papillary thyroid carcinoma, 22 benign nodules, 1 follicular adenoma, 1 case of Hashimoto’s thyroiditis, 1 oncocytic adenoma, and 1 case of anaplastic thyroid carcinoma (Table 6).

When comparing the diagnostic yields of FNAC and CNB, the sensitivity, specificity, and accuracy of FNAC were 100% (95% confidence interval [CI], 86.7–100%), 42.9% (95% CI, 1.0–81.6%), and 87.9% (95% CI, 71.8–96.6%), respectively. For CNB, the sensitivity, specificity, and accuracy were 100% (95% CI, 78.2–100%), 70.0% (95% CI, 34.8–93.3%), and 88.0% (95% CI, 68.8–97.5%), respectively, indicating a higher specificity in the CNB group (Table 7).

### 3.5. Post-Procedural Complications

No post-procedural complications, such as hematoma due to vascular injury, voice changes due to recurrent laryngeal nerve damage, infection, subcutaneous emphysema, or hemoptysis due to tracheal injury, were reported for both FNAC and CNB.

## 4. Discussion

In this study, CNB demonstrated a higher frequency of conclusive results than FNAC in primary examinations, consistent with the findings of Suh et al. [21]. Although Bethesda Category IV (follicular neoplasm/suspicious for follicular neoplasm) is pathologically indeterminate and cannot discriminate between a follicular adenoma and a follicular carcinoma without evaluation of the entire tumor capsule, as stated in the 2019 Practice Guidelines for Thyroid Core Needle Biopsy of the Korean Thyroid Association, in surgical departments these findings routinely lead to surgical management for diagnostic or therapeutic purposes. Therefore, in this study, we classified Category IV as ‘conclusive’ to reflect its role in guiding clinical decision-making rather than a confirmed histopathological distinction [23]. This observation was particularly evident in patients who underwent both CNB and FNAC simultaneously, likely because of the greater quantity of tissue obtained with CNB [10]. Furthermore, as indicated by Hong et al., CNB can analyze both cytological and histological features, which may account for the differing results compared to FNAC [25]. The higher rate of conclusive results with CNB in primary examinations highlights its potential clinical utility in guiding decisions for surgery or monitoring.

When comparing nodules that underwent secondary FNAC or CNB following an initially inconclusive FNAC, the rate of conversion to a conclusive diagnosis was significantly higher in the secondary CNB group. This finding aligns with previous studies supporting the utility of CNB as a secondary diagnostic procedure [17]. Although both FNAC and CNB demonstrated similar sensitivity and diagnostic accuracy in this study, CNB exhibited higher specificity. Moreover, no complications were reported for either procedure, suggesting that CNB may offer a higher diagnostic yield than FNAC when performed safely and appropriately.

According to the 2017 guidelines from the Korean Society of Thyroid Radiology, the indications for CNB include: (1) non-diagnostic results from FNAC, (2) indeterminate nodules from FNAC, and (3) suspected lymphoma, anaplastic thyroid carcinoma, medullary thyroid carcinoma, or metastatic cancer to the thyroid [26]. The guidelines currently state that there is insufficient evidence to replace FNAC with CNB as an initial diagnostic procedure for thyroid nodules, emphasizing the need for further research on CNB’s efficacy. Additionally, future studies should consider a cost–benefit analysis of FNAC and CNB, given the increased number of visits, costs, and diagnostic delays associated with indeterminate results.

The strength of this study lies in its design, which concurrently analyzed primary and secondary examination results within the same patient cohort, rather than focusing solely on nodules with initially non-diagnostic FNAC results. This comprehensive approach evaluates the complementarity of FNAC and CNB, provides evidence for selecting diagnostic methods, and aids in planning the diagnosis and treatment of thyroid nodules. These findings may also guide the design of future research.

The limitations of this study include the potential selection bias inherent in its retrospective design, particularly as cases in which CNB was performed following primary FNAC may have had a higher suspicion of malignancy or follicular neoplasm. Moreover, although FNAC was mandated for specific nodule types (e.g., large cystic nodules), in certain cases, the selection of the diagnostic modality was influenced by patient preference. This real-world practice may have introduced additional selection bias beyond what is reflected by baseline demographics. For instance, patients with nodules exhibiting high-suspicion ultrasonographic features or with prior inconclusive FNAC results may have been more inclined to undergo CNB, whereas those with small, low-suspicion nodules may have favored FNAC. Such factors could have altered the baseline characteristics of the two groups and should be considered when interpreting our findings. The relatively small sample size and the presence of some patients without final surgical pathology due to drop-out are also limitations. Additionally, CNB requires operator proficiency, and results for nodules smaller than 1 cm or located posteriorly in the thyroid may vary among practitioners [25]. Therefore, large-scale, multicenter prospective studies are necessary to further validate our findings and establish cost-effectiveness. Although CNB demonstrated a higher frequency of conclusive results than FNAC in our cohort, its application should be carefully tailored to appropriate clinical contexts. During the study period, FNAC and CNB were selected based on both specific clinical circumstances and patient preference. Nevertheless, in our institution, CNB has typically been performed in nodules with repeated inconclusive FNAC results, high-suspicion ultrasonographic features (K-TIRADS 4 or 5), suspected lymphoma or anaplastic thyroid carcinoma, or in cases in which FNAC was technically challenging (e.g., deeply located or posteriorly situated nodules). Clearly defining these indications underscores the appropriate clinical context for CNB use and may help avoid unnecessary procedures.

## Figures and Tables

**Table 1:** Patient demographic and ultrasonographic findings.

Characteristics	FNAC Group (*n* = 196)	CNB Group (*n* = 93)	*p* Value
Sex			0.644
Male	64 (32.7)	28 (30.1)	
Female	132 (67.3)	65 (69.9)	
Age (years)	54.3 ± 15.4	56.9 ± 15.20	0.179
No. of nodule	228	110	
Nodule size (cm)	1.80 ± 0.61	1.72 ± 1.10	0.660
US pattern (No. of nodules)			0.054
Low suspicion (K-TIRADS 3)	99 (43.4)	50 (45.5)	
Intermediate suspicion (K-TIRADS 4)	84 (36.9)	28 (25.5)	
High suspicion (K-TIRADS 5)	45 (19.7)	32 (29.0)	

Data are presented as mean ± standard deviation, number (%). US, Ultrasonography; FNAC, Fine needle aspiration cytology; CNB, Core needle biopsy; K-TIRADS, Korean Thyroid Imaging Reporting and Data System.

**Table 2:** Primary examination results.

Characteristics	No. of Nodules (%)		*p* Value
	FNAC Group (*n* = 228)	CNB Group (*n* = 110)	
Diagnostic category ^(a)^			
I	68 (29.8)	3 (2.7)	
II	92 (40.4)	58 (52.7)	
III	45 (19.7)	28 (25.5)	
IV	1 (0.4)	2 (1.8)	
V	19 (8.3)	9 (8.2)	
VI	3 (1.3)	10 (9.1)	
Integrated result			<0.001 *
Conclusive (II + IV + V + VI)	115 (50.4)	79 (71.8)	
Inconclusive (I + III)	113 (49.6)	31 (28.2)	

FNAC, Fine needle aspiration cytology; CNB, Core needle biopsy. ^(a)^ Diagnoses were classified into six categories according to the Bethesda system and the 2019 Practice Guidelines for Thyroid Core Needle Biopsy of the Korean Thyroid Association. A conclusive result was defined as Category II (benign), Category IV (follicular neoplasm/suspicious for follicular neoplasm), Category V (suspicious for malignancy), and Category VI (malignancy). These categories represent diagnoses that provide sufficient information to determine further management, such as surgery or observation, without the need for additional diagnostic testing. An inconclusive result was defined as Category I (non-diagnostic) and Category III (atypia of undetermined significance/follicular lesion of undetermined significance [AUS/FLUS]). These categories indicate that the diagnosis remains uncertain and additional diagnostic procedures are required to achieve a definitive result. Statistical analyses were conducted with a significance threshold of *p* < 0.05. * *p* < 0.05.

**Table 3:** Cases of patients with concurrent FNAC and CNB as an initial diagnostic procedure.

Case	Sex	Age (Years)	Size of Nodule (cm)	K-TIRADS	FNAC Result ^(a)^	CNB Result ^(a)^	Surgical Pathology Result
1	F	75	1.38	3	II	III	NA ^(b)^
2	M	33	5.56	3	I	II	Nodular hyperplasia
3	F	39	2.19	5	III	V	Papillary thyroid carcinoma
4	F	33	4.44	5	I	II	Nodular hyperplasia
5	M	34	4.92	5	I	VI	NA ^(b)^
6	F	57	0.57	4	I	V	Papillary thyroid carcinoma
7	M	69	1.6	5	I	VI	Papillary thyroid carcinoma
8	M	52	0.7	4	I	II	NA ^(b)^

K-TIRADS, Korean Thyroid Imaging Reporting and Data system; FNAC, Fine needle aspiration cytology; CNB, Core needle biopsy; F, Female; M, Male; NA, not available. ^(a)^ Diagnoses in this study were classified according to the six categories outlined in the Bethesda system and the 2019 Practice Guidelines for Thyroid Core Needle Biopsy of the Korean Thyroid Association. ^(b)^ The reasons for unavailable surgical pathology results in this study included the referral of patients to other hospitals based on their preference and the absence of planned surgeries due to benign diagnoses or low suspicion for malignancy based on imaging and biopsy findings.

**Table 4:** Results of nodules subjected to FNAC as primary and secondary examinations.

First Integrated Result (Diagnostic Category) ^(a)^	Second Integrated Result (Diagnostic Category) ^(a)^		Total	*p* Value
	Inconclusive (I + III)	Conclusive (II + IV + V + VI)		
Inconclusive (I + III)	8 (44.4)	6 (33.3)	14 (77.8)	0.289
Conclusive (II + IV + V + VI)	2 (11.1)	2 (11.1)	4 (22.2)	
Total	10 (55.6)	8 (44.4)		

Data are presented as number (%). ^(a)^ Diagnoses in this study were categorized based on the six categories outlined in the Bethesda system and the 2019 Practice Guidelines for Thyroid Core Needle Biopsy of the Korean Thyroid Association. A conclusive result was defined as Category II (benign), Category IV (follicular neoplasm/suspicious for follicular neoplasm), Category V (suspicious for malignancy), and Category VI (malignancy). These categories provide sufficient diagnostic information to guide clinical management, such as observation or surgical intervention. An inconclusive result was defined as Category I (non-diagnostic) and Category III (atypia of undetermined significance/follicular lesion of undetermined significance [AUS/FLUS]). These categories indicate diagnostic uncertainty, often necessitating additional diagnostic procedures, such as repeat FNAC, CNB, or surgical biopsy, to achieve a definitive diagnosis.

**Table 5:** Results of nodules subjected to FNAC as primary and CNB as secondary examinations.

First Integrated Result (Diagnostic Category) ^(a)^	Second Integrated Result (Diagnostic Category) ^(a)^		Total	*p* Value
	Inconclusive (I + III)	Conclusive (II + IV + V + VI)		
Inconclusive (I + III)	8 (42.1)	8 (42.1)	16 (84.2)	0.008 *
Conclusive (II + IV + V + VI)	0	3 (15.8)	3 (15.8)	
Total	8 (42.1)	11 (57.9)		

Data are presented as number (%). ^(a)^ Diagnoses in this study were categorized according to the six categories defined by the Bethesda system and the 2019 Practice Guidelines for Thyroid Core Needle Biopsy of the Korean Thyroid Association. A conclusive result was defined as Category II (benign), Category IV (follicular neoplasm/suspicious for follicular neoplasm), Category V (suspicious for malignancy), and Category VI (malignancy). These categories provide sufficient diagnostic information to guide clinical management, such as observation, surgical intervention, or treatment planning. An inconclusive result was defined as Category I (non-diagnostic) and Category III (atypia of undetermined significance/follicular lesion of undetermined significance [AUS/FLUS]). These categories indicate diagnostic uncertainty and often require additional diagnostic procedures, such as repeat FNAC, CNB, or surgical biopsy, to establish a definitive diagnosis. All statistical analyses were conducted with a significance threshold of *p* < 0.05. * *p* < 0.05.

**Table 6:** Final histopathological results.

Characteristics	Number of Nodules
Benign	
Nodular hyperplasia	22 (35.4)
Follicular adenoma	1 (1.6)
Hürthle cell adenoma	1 (1.6)
Hashimoto’s thyroiditis	1 (1.6)
Malignant	
Papillary thyroid carcinoma	36 (58.0)
Poorly differentiated thyroid carcinoma	1 (1.6)

Data are presented as number (%).

**Table 7:** Comparison of diagnostic yields between fine needle aspiration cytology and core needle biopsy based on post-surgical definitive histopathological results.

	% (95% CI)		
	Sensitivity	Specificity	Accuracy
FNAC	100 (86.7–100)	42.9 (1.0–81.6)	87.9 (71.8–96.6)
CNB	100 (78.5–100)	70.0 (34.8–93.3)	88.0 (68.8–97.5)

FNAC, Fine needle aspiration cytology; CNB, Core needle biopsy; CI, Confidence interval.

## Data Availability

The data presented in this study are available on request from the corresponding author due to restrictions on patient privacy.

## Abbreviations

The following abbreviations are used in this manuscript:

AUS	atypia of undetermined significance
CI	confidence interval
CNB	core needle biopsy
FLUS	follicular lesion of undetermined significance
FN	follicular neoplasm
FNAC	Fine-needle aspiration cytology
K-TIRADS	Korean Society of Thyroid Radiology
SFN	suspicious for follicular neoplasm
